# Novel insights into pathomechanisms of retinal neuronal degeneration and reactive gliosis in a murine model of G_M1_-gangliosidosis

**DOI:** 10.1038/s41598-025-15639-9

**Published:** 2025-08-13

**Authors:** Lorna Jubran, Rouven Wannemacher, Anastasiia Ulianytska, Ingo Gerhauser, Wolfgang Baumgärtner, Eva Leitzen

**Affiliations:** 1 Department of Pathology, University of Veterinary Medicine Hannover, Foundation, Hannover, Germany; 2 Center for Systems Neuroscience, Hannover Graduate School for Neurosciences, Infection Medicine, and Veterinary Sciences (HGNI), Hannover, Germany

**Keywords:** G_M1_-gangliosidosis, G_M1_ accumulation, Retinal pathology, Lysosomal storage disease, Müller cells, Microglia activation, Retinal gliosis, Eye diseases, Metabolic disorders, Neurological disorders

## Abstract

G_M1_-gangliosidosis is a lysosomal storage disease characterized by the accumulation of G_M1_ ganglioside in neurons, including retinal ganglion cells (RGCs). Although vision impairment and retinal degeneration are well-known manifestations in humans, knowledge about the underlying mechanism of these lesions is limited. Pathological retinal changes in a *Glb1* knockout (*Glb1*^−/−^) mouse model were assessed using immunohistochemistry, immunofluorescence and transmission electron microscopy in 4- and 7-month-old wild type and *Glb1* knockout (*Glb1*^−/−^) mice. Increased numbers of glial fibrillary acidic protein (GFAP) positive Müller cells (MCs) were detected in *Glb1*^*−/−*^ mice both at 4 and 7 months of age, indicating glial reactivity. A transient increase in glutamine synthetase (GS) positive MCs at 4 months of age with a subsequent decrease by 7 months, most likely related to reduced expression of GS, was also observed. Immunohistochemistry revealed loss of RGCs, storage material accumulation and decreased BRN3A expression in 7-month-old *Glb1*^−/−^ mice. Increasing numbers of Iba1-positive microglia/macrophages were detected in *Glb1*^−/−^ mice at both time points. Microglia/macrophages showed migration towards the inner retinal layers and acquired a morphological phenotype that indicates activation. The present study shows that the murine G_M1_ model used in this study is suitable for investigating ocular changes in lysosomal storage diseases.

## Introduction

G_M1_-gangliosidosis is a rare inherited metabolic neurodegenerative disease caused by a deficiency of β-galactosidase (β-gal)^1^. The enzyme deficiency leads to progressive storage of G_M1_ ganglioside (G_M1_) and its derivates, particularly within lysosomes of neuronal tissue^2–4^. In humans, the disease is classified into three clinical subtypes: the infantile (type 1, OMIM: 230500), the late infantile (juvenile; type 2, OMIM: 230600), and the adult form (adult; type 3, OMIM: 230650). The clinical symptoms in human G_M1_-gangliosidosis vary depending on the age of onset and the specific disease phenotype^4^.

Various naturally occurring and genetically modified animal models have been utilized for G_M1_ research^5–7^. A comparable naturally occurring genetic defect for G_M1_-gangliosidosis in animals has also been reported in cats, dogs, cattle, sheep, emus, and American black bears^8–13^. The first transgenic mice were reported in 1997 by Hahn et al. and Matsuda et al. (1997)^14,15^. These models closely resemble the infantile/juvenile form of the disease, exhibiting pronounced neuronal loss and neuroinflammation^14,15^. More recent murine models have been developed using TALEN and CRISPR/Cas9 technologies. In 2019, Przybilla et al. introduced a knockout model with a deletion in exon 8 of the *Glb1* gene, which resulted in neurocognitive impairment^16^. In 2020, Eikelberg et al. reported a new *Glb1*^−/−^ mouse model that mimics the adult form of G_M1_-gangliosidosis with delayed onset of observable symptoms and limited lesion distribution^17^. More recently, Liu et al. generated a transgenic mouse model characterized by a human missense mutation in exon 14 that mimics the late-infantile subtype. This model exhibits pronounced motor function impairment and extensive microgliosis^18^.

Despite the extensive research conducted on the pathomechanism of G_M1_-gangliosidosis, ocular pathology associated with G_M1_ accumulation remains insufficiently characterized^19,20^. Ocular manifestations in human patients may include visual impairment, corneal opacities, tortuous retinal vessels, retinal haemorrhages and optic atrophy^19^. Notably, a cherry-red macula is detected in about half of the cases^19^. This significant fundoscopic finding represents a hallmark feature in numerous ganglioside catabolism disorders^21^. Pathological changes in ocular structures during G_M1_-gangliosidosis have already been described in cats, dogs, and calves^22–25^. However, retinal changes have not yet been investigated in rodent models of G_M1_ gangliosidosis.

Pathological processes within the retina, including neuronal damage, are often accompanied by glial cell activation and reactive gliosis^26,27^. The mammalian retina contains two main categories of macroglial cells, astrocytes and Müller cells (MCs), alongside resident microglia^28^. MCs belong to a special subgroup of glial cells of the central nervous system (CNS), also known as aldynoglia. They appear as radially oriented cells whose processes extend across the entire thickness of the retina from the inner limiting membrane (ILM) to the distal end of the outer nuclear layer (ONL)^26^. In the ILM, they form structures called endfeet that wrap around axons and somas of retinal ganglion cells (RGCs)^26^. Consequently, MCs provide structural support and play a pivotal role in maintaining retinal homeostasis, including involvement in the organization of the blood-retinal barrier^29–31^. Upon activation, MCs exhibit reaction patterns similar to other glial cells of the CNS and peripheral nervous system (PNS). Under pathological conditions, they show an upregulation of glial fibrillary acidic protein (GFAP), which is also observed in astrocytes as well as PNS satellite glial cells (SGCs) of the dorsal root ganglia (DRG)^32–34^. Another shared feature of MCs, astrocytes and SGCs is the continuous expression of glutamine synthetase (GS) under physiological circumstances^30,35^. The expression of this enzyme is crucial for the regulation and recycling of excess glutamate from neuronal synapses, ensuring effective neurotransmitter recovery^36,37^. Retinal microglia are predominantly located in the inner and outer plexiform layers (IPL, OPL) in the physiological state^38^. Comparable to brain microglia, their cellular phenotypes can change depending on their activation status^39,40^. Resting, inactivated microglia show ramified processes^39–41^. Upon activation, a change in morphology towards enlarged, partly amoeboid cell bodies with shortening and thickening of cellular processes towards small pseudopodia can be observed^39,40,42^. A profound characterization of these morphological and functional changes is not only critical for understanding retinal disease mechanisms but also for the development of potential therapeutic approaches^43^.

The present study investigates the phenotypic and morphological changes of retinal ganglion cells and retinal glial cells in a late-onset *Glb1*^−/−^ mouse model during the course of G_M1_-gangliosidosis. The aim is to characterise neurodegenerative changes and the subsequent reaction pattern of retinal glial cells, with a focus on MCs and microglial cells.

## Results

### Müller cells upregulate GFAP in 4- and 7-months-old *Glb1*^−/−^ mice

**Fig. 1:**
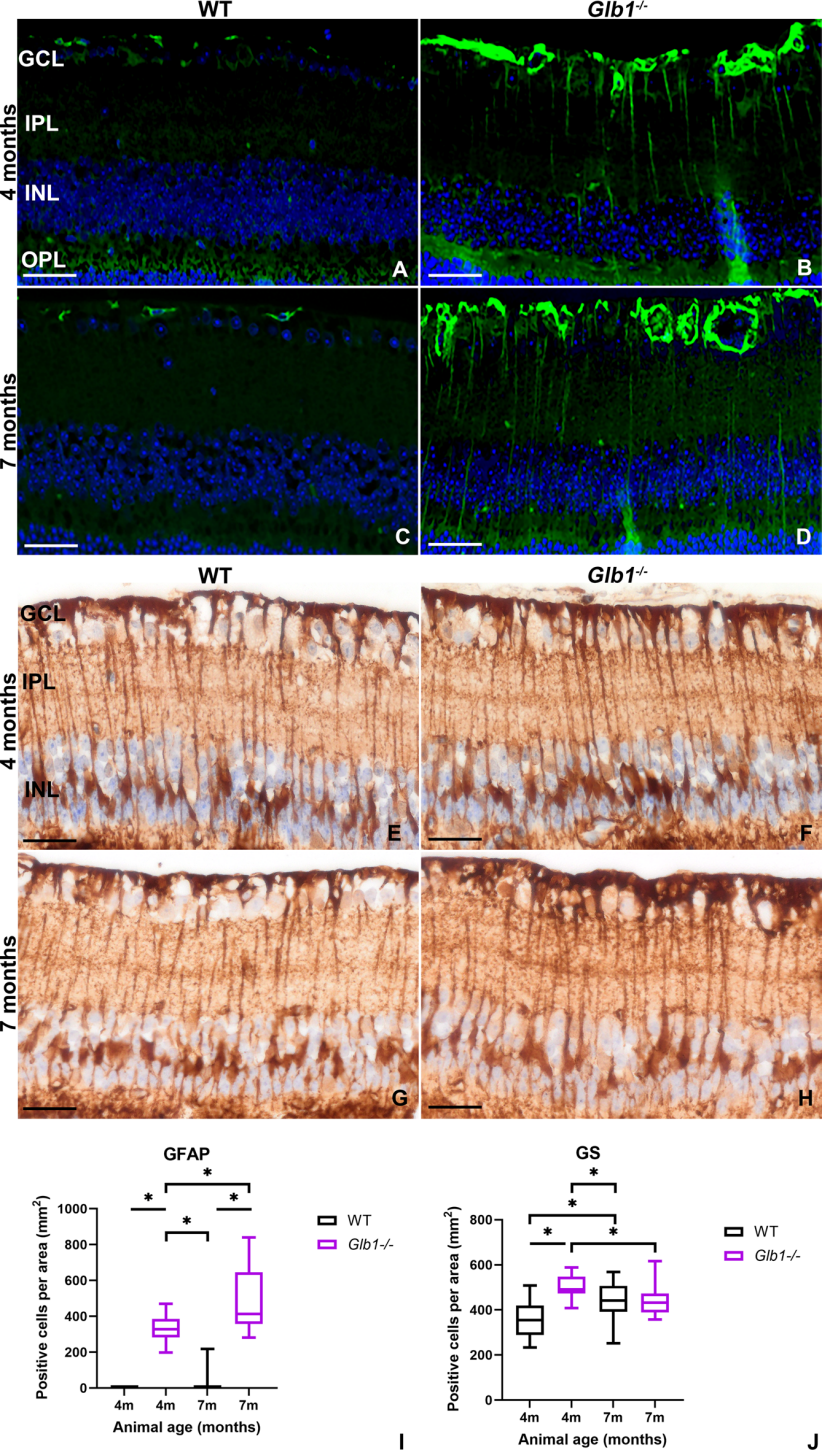
Immunofluorescence (**A–D**) and immunohistochemistry (**E–H**) targeting glial fibrillary acidic protein (GFAP, green) and glutamine synthetase (GS) in Müller cells (MCs) in 4- and 7-month-old wild type (WT) and *Glb1* knockout (*Glb1*^−/−^) mice. WT mice show weak GFAP positive signals only within uppermost retinal layers at both time points investigated, most likely in the endfeet of MCs and perivascular astrocytes (**A**,** C**). Within 4 and 7-month-old *Glb1*^−/−^ mice, strong GFAP positive signals can be detected also labeling the cell bodies of MCs, extending into deeper retinal layers (**B**,** D**). Nuclei are counterstained with bisbenzimide (blue). 4 and 7-month-old WT and *Glb1*^−/−^ mice show high numbers of MCs immunopositive for the MC specific marker GS. Quantification of GS positive MCs reveals an increase of GS positive cells in *Glb1*^−/−^ mice at 4 months of age (**F**) compared to WT animals (**E**). No difference was detected between WT and *Glb1*^−/−^ mice in 7-month-old animals (**H**), probably due to downregulation of GS in MCs as result of progressive neuronal loss. Scale bars: 20 μm. Statistical analysis of GFAP and GS expression in retinal MCs (**I**,**J**). Graphs display box and whisker plots. Significant differences between groups were detected by Kruskal-Wallis test followed by Dunn’s-Bonferroni procedure; *p ≤ 0.05; *n* = 5. GCL, ganglion cell layer; IPL, inner plexiform layer; INL, inner nuclear layer; OPL, outer plexiform layer.

Immunofluorescence (IF) staining of retinal tissue for GFAP (Fig. 1A–D) demonstrated positive signals in WT animals only within innermost retinal layers, most likely detecting MC end feet and perivascular astrocytes (Fig. 1A,C). Increasing GFAP-positive signals in MCs, which also stain vertically orientated cell bodies (Fig. 1B,D), could be detected in *Glb1*^−/−^ mice with significant differences to WT animals from the age of 4 months. An increasing number of GFAP-positive MCs was detected in knockout mice (Fig. 1I) until the end of the investigation period, indicating an upregulation of GFAP in response to ongoing RGC damage in this *Glb1*^−/−^ mouse model^17,34^. This raised the question, whether MCs also showed changes in other cell type specific markers.

### Early increase and subsequent loss of glutamine synthetase positive Müller cells in *Glb1*^−/−^ mice

Immunohistochemistry (IHC) targeting GS (Fig. 1E–H) and subsequent quantification of positive cells revealed significant differences in the number of GS-positive MCs between WT and *Glb1*^−/−^ mice starting at 4 months of age (Fig. 1J). A significant increase was also detected between 4-month-old and 7-month-old WT mice, indicating age-related changes. Interestingly, 7-month-old *Glb1*^−/−^ mice showed no significant difference in GS-positive MCs compared to their age-matched WT counterparts but a significant decrease when compared to 4-month-old *Glb1*^−/−^ mice. In order to determine the possible interactions of these partly transient phenotypic changes with neuronal changes in more detail, the RGCs were examined more closely.

### Neuronal loss is accompanied by increased accumulation of storage material and decreased expression of transcription marker BRN3A in 7-month-old *Glb1*^−/−^ mice

**Fig. 2:**
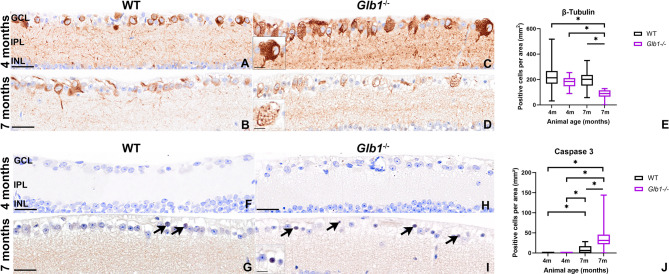
Immunohistochemistry (IHC) targeting βIII-tubulin for visualization of retinal ganglion cells (RGCs) (**A–D**) and Caspase 3 for quantification of neuronal apoptosis (**F–I**) in wild type (WT) mice as well as *Glb1* knockout (*Glb1*^−/−^) mice at 4- and 7-month-old. Positive cytoplasmic staining can be detected in retinal ganglion cells (RGCs) with prominent intracytoplasmic vacuoles especially in 7-month-old *Glb1*^−/−^ mice (**A–D**). Quantification of RGCs revealed that 7-month-old *Glb1*^−/−^ mice show a significant reduction of neuronal cells compared to all other groups, including 4-month-old *Glb1*^−/−^ mice (**E**). Using IHC targeting cleaved caspase-3 a weak intranuclear signal was detected especially in 7-month-old *Glb1*^−/−^ RGCs (**I**, arrows). Within 4-month-old WT (**F**) and *Glb1*^−/−^ mice (**H**) no immunopositive reaction was observed. Within the retina of 7-month-old WT mice (**G**, arrows), a mildly increased number of ganglion cells with intranuclear positive results (arrow) could be detected (**J**). 7-month-old *Glb1*^−/−^ mice show a significant increase in caspase 3-positive RGCs (arrows; insert) compared to all other investigation groups (**J**). Scale bars: 20 μm; Insert (**C**,**D**,**I**): 10 μm. Graphs display box and whisker plots. Significant differences between groups were detected by Kruskal–Wallis test followed by Dunn’s–Bonferroni procedure; *p ≤ 0.05; *n* = 5. GCL, ganglion cell layer; IPL, inner plexiform layer; INL, inner nuclear layer.

IHC targeting βIII-tubulin, a pan-neuronal marker, was used for overall quantification of neuronal cells of the ganglion cell layer (Fig. 2A–D). Data (Fig. 2E) indicate a significant loss of neurons in 7-month-old *Glb1*^−/−^ mice when compared with all other experimental groups, including WT mice at 4 and 7 months of age but also *Glb1*^−/−^ mice at 4 months. This indicates a progressive loss of neuronal cells, especially at late time points at an advanced stage of the disease.

In addition, IHC targeting cleaved caspase-3 to screen for neuronal apoptosis (Fig. 2F–I) was used. No caspase 3-positive cells were detected in 4-month-old WT and *Glb1*^−/−^ mice (Fig. 2F,H). Within WT mice at 7 months of age, few immunopositive neurons were detected (Fig. 2G), while comparable staining results were frequently seen in neurons of 7-month-old *Glb1*^−/−^ mice (Fig. 2I). RGCs showed weak, brownish, punctate-like, nuclear staining^44^. Cells appeared partially shrunken with condensed chromatin, suggesting apoptosis based on IHC staining and morphology. 7-month-old WT and *Glb1*^−/−^ mice showed significantly increased numbers of positive cells when compared to 4-month-old animals. Moreover, *Glb1*^−/−^ mice at 7 months of age showed a significant increase in apoptotic ganglion cells compared to age-matched WT animals (Fig. 2J).

**Fig. 3:**
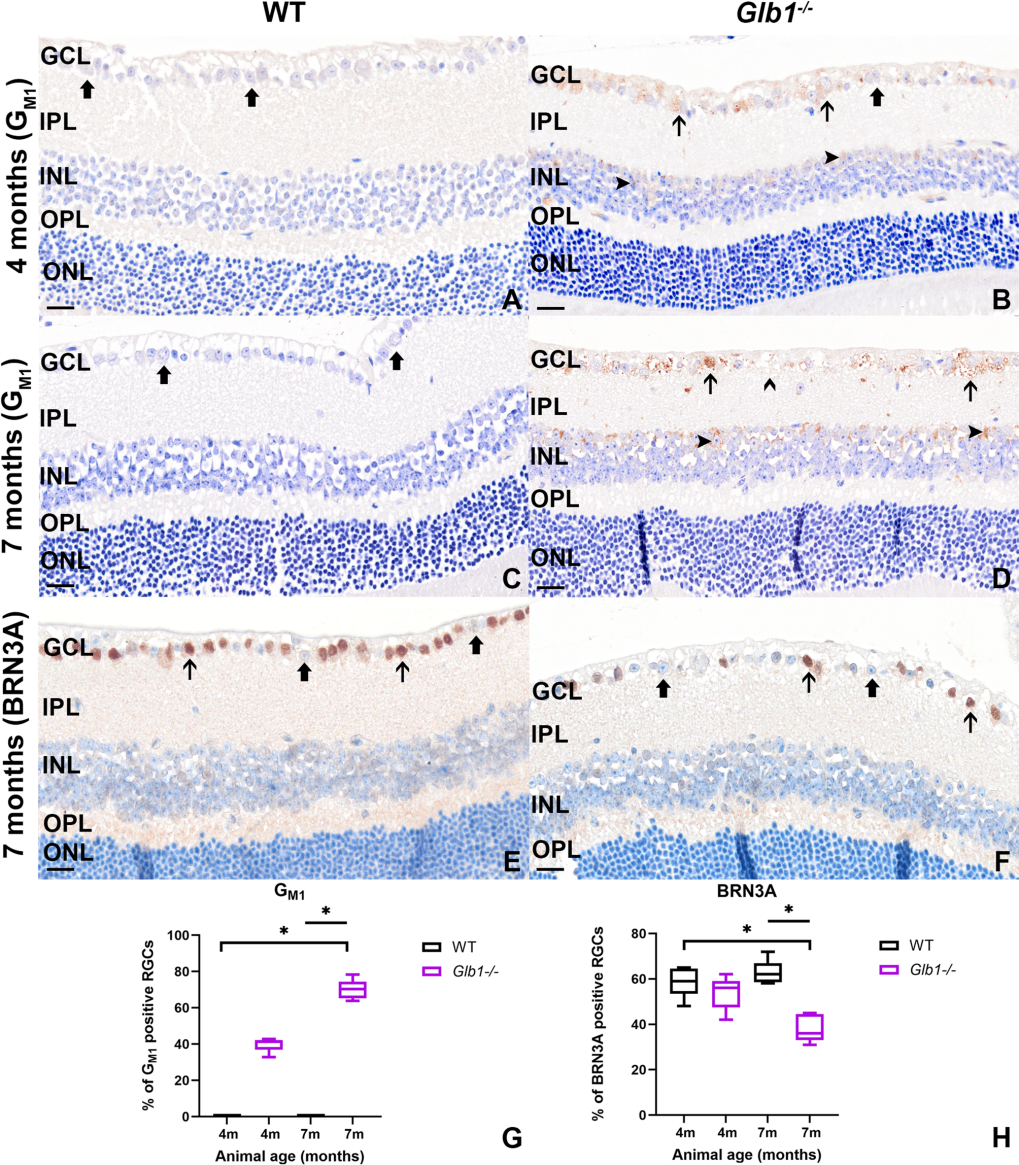
Representative images of immunohistochemistry targeting storage material (G_M1_) within the retinas of 4- (**A**) and 7-month-old (**C**) wild type (WT) as well as 4- (**B**) and 7-month-old (**D**) *Glb1* knockout (*Glb1*^−/−^) mice as well as BRN3A-positive neurons of the GCL in 7-month-old WT (**E**) and *Glb1*^−/−^ (**F**) mice. Storage material is not detected in neurons of the ganglion cell layer in WT mice at both time points (**A**,** C**; thick arrows). Several neurons with brownish, granular, intracytoplasmic signals can be observed in 4- and 7-month old *Glb1*^−/−^ mice (**B**,**D**; thin arrows). Some neurons without obvious intracytoplasmic signals can be observed, especially within 4-month-old *Glb1*^−/−^ mice (**B**; thick arrow). In 7-month-old animals there is loss of visible neuronal nuclei (**D**; open arrowhead). Moreover, within the inner nuclear layer, scant (**B**; closed arrowheads) and more pronounced (**D**; closed arrowheads) accumulation of storage material can be detected. No positive signals were detected within the outer nuclear layer. **A-F**: scale bars: 20 μm. **G**,**H**: Graphs display box and whisker plots. Significant differences between groups were detected by Kruskal–Wallis test followed by Dunn’s–Bonferroni procedure; * *p* ≤ 0.05; *n* = 5. GCL, ganglion cell layer; IPL, inner plexiform layer; INL, inner nuclear layer; OPL, outer plexiform layer; ONL, outer nuclear layer.

In parallel, GCL neurons with intracytoplasmic accumulation of G_M1_ storage material were quantified as percentage of all GCL neurons examined (Fig. 3). WT animals showed no positive signals (Fig. 3A,C) while neurons with granular to vacuolated signals were detected in 4- and 7-month-old *Glb1*^−/−^ mice (Fig. 3B,D). Statistical analysis revealed significant differences between both WT groups and 7-month-old knockout mice (Fig. 3G). Interestingly, cells within the apical layer of the INL showed positive signals in *Glb1*^−/−^ mice, indicating storage material accumulation also within this cell population, while no signals were detected within the ONL.

To further investigate RGC integrity, IHC for BRN3A, a transcription factor specifically expressed in RGCs, was conducted. BRN3A-positive cells were clearly detectable in the GCL of both WT and *Glb1*^−/−^ mice (Fig. 3E,F). Quantification and statistical analysis revealed a significant reduction of BRN3A positive RGCs in 7-month-old *Glb1*^−/−^ mice (Fig. 3F,H). BRN3A cell counts remained stable in WT mice between 4 and 7 months, indicating a progressive loss of BRN3A positivity in *Glb1*^−/−^ mice.

### Disease progression is associated with increased numbers, directed migration and morphologic changes in Iba1-positive cells in *Glb1*^−/−^ mice

**Fig. 4:**
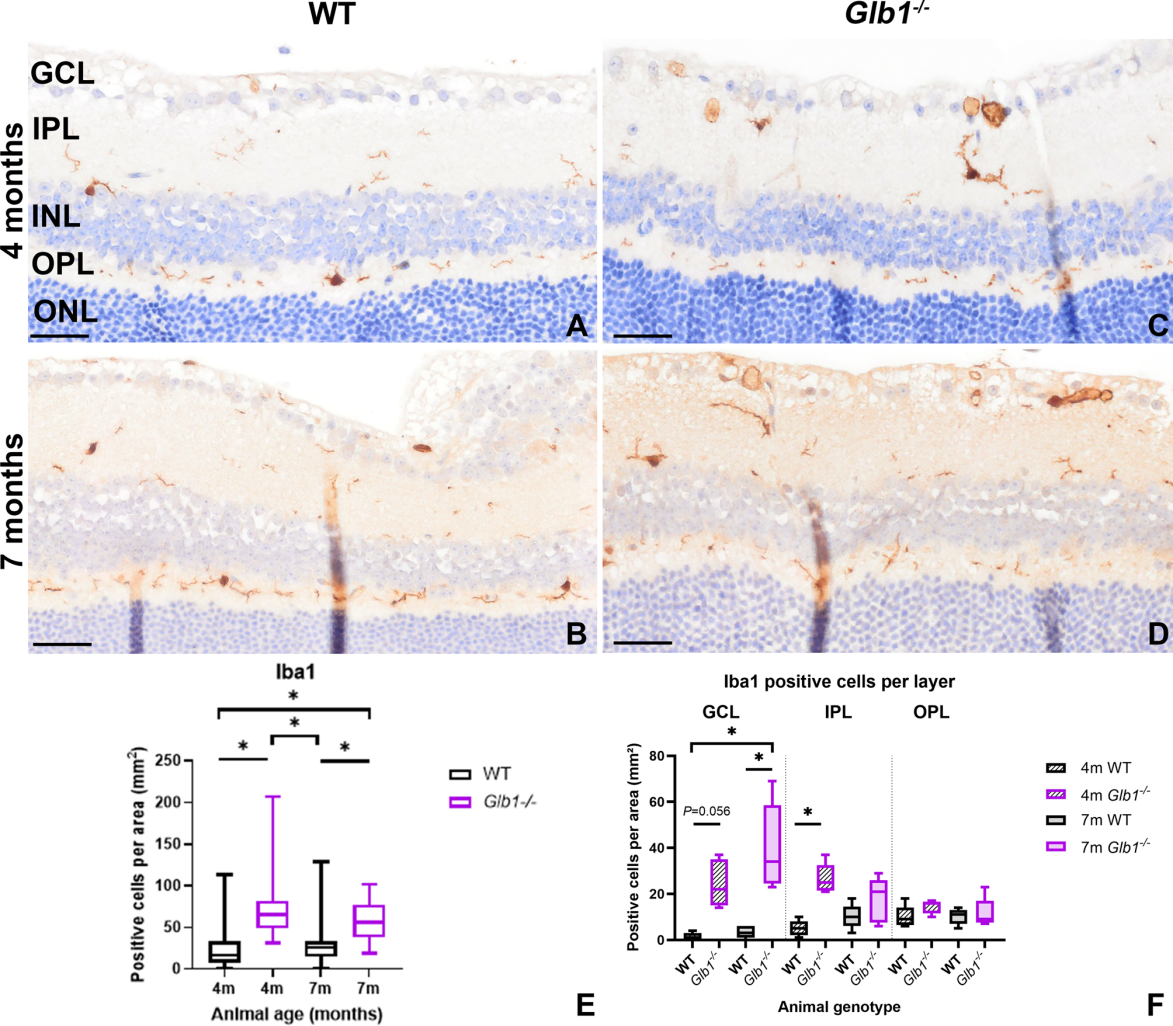
Immunohistochemistry targeting Iba1-positive cells within retinal layers. Wild type (WT) animals show low numbers of Iba1-positive cells in 4- (**A**) and 7-month-old mice (**B**). Increased numbers of Iba1-positive cells can be detected in 4- (**C**) and 7-(**D**) month-old *Glb1* knockout (*Glb1*^−/−^) mice, especially in the ganglion cell layer (GCL) and inner plexiform layer (IPL). Scale bars: 50 μm. Quantification of total numbers of retinal Iba1 positive cells revealed significant differences between *Glb1*^−/−^ and WT mice (**E**). Quantification of Iba1-positive cells according to different retinal layers shows a predominant increase in innermost retinal layers (GCL, IPL) compared to, for example, the outer plexiform layer (OPL) (**F**). Graphs display box and whisker plots. Significant differences between groups were detected by Kruskal-Wallis test followed by Dunn’s-Bonferroni procedure; * *p* ≤ 0.05; *n* = 5. INL, inner nuclear layer. Folds within the inner layers of retinal tissue have been digitally lightened.

**Fig. 5:**
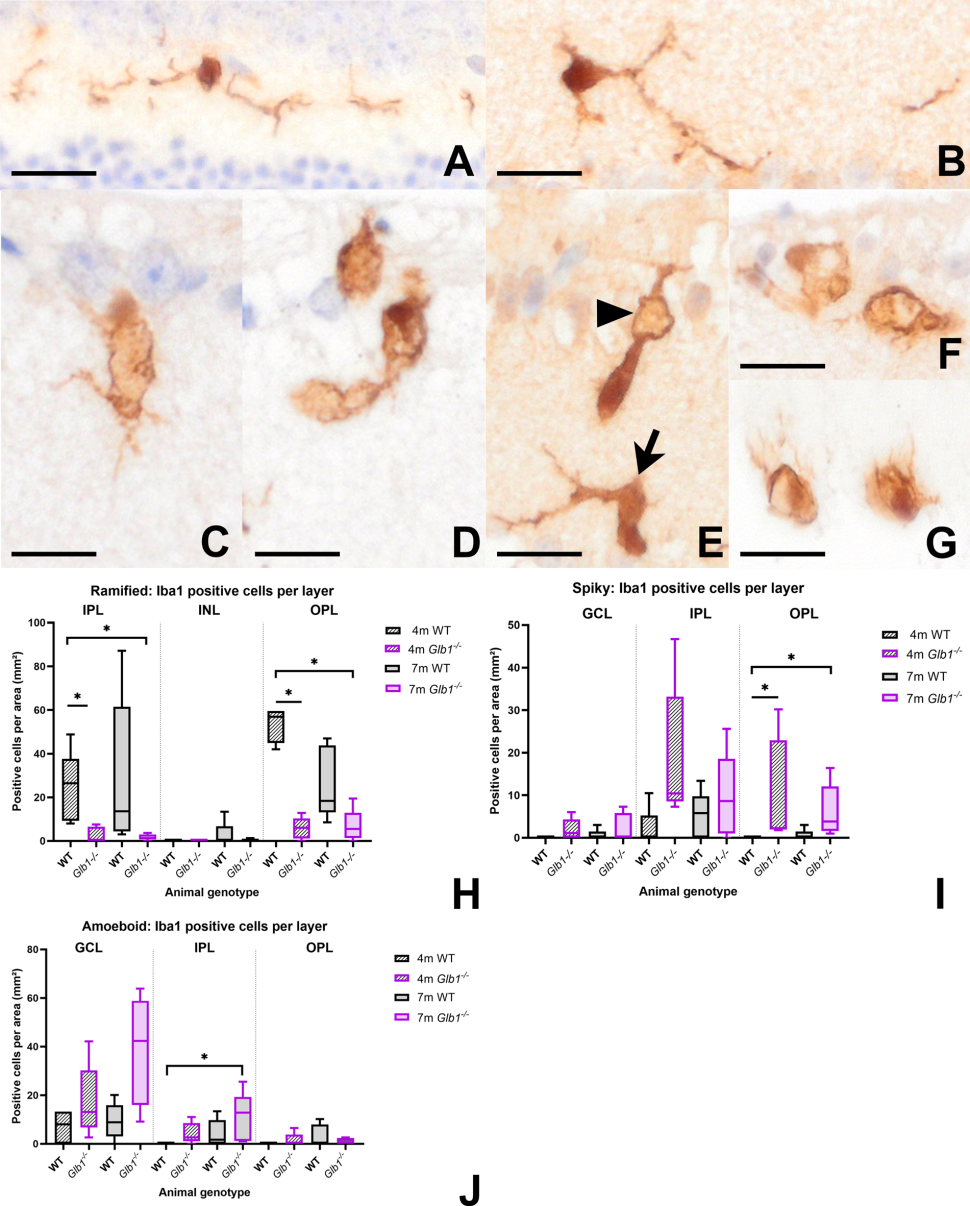
Representative images of immunohistochemistry targeting Iba1 demonstrating distinct microglia/macrophage morphologies within different retinal layers. Ramified microglia, characterized by long and thin cellular processes around a small, distinct soma, were mostly located within OPL and IPL of wild type (WT) mice. A ramified morphology indicates a non-activated, resting state (**A**,** B**), which is most abundant in the OPL and IPL of WT mice (**H**). Activation of microglia/macrophages in *Glb1* knockout (*Glb1*^−/−^) mice leads to shortening and thickening of processes resulting in a spiky morphology and enlarged, more irregular cell bodies. Spiky Iba1-positive cells (**C**, **E**: arrow, **G**) were mostly detected in the OPL of *Glb1*^−/−^ mice (**I**). Amoeboid microglia/macrophages display a roundish morphology with very short or even absent processes as well as an increased diameter of the soma (**D**,** E**: arrowhead, **F**). This phenotype is mostly detected in the IPL layers of *Glb1*^−/−^ mice (**J**). Scale bars: 10 μm. Graphs display box and whisker plots. Significant differences between groups were detected by Kruskal-Wallis test followed by Dunn’s-Bonferroni procedure; *p ≤ 0.05; *n* = 5. IPL, inner plexiform layer; OPL, outer plexiform layer.

Whether these degenerative changes are associated with a secondary reaction of phagocytic cells was addressed by studying the role of ionized calcium-binding adapter molecule 1 (Iba1) positive cells (microglia/macrophages). Iba1-positive cells were detected in both WT and *Glb1*^−/−^ mice at 4 and 7 months of age (Fig. 4A–D). Statistical analysis of Iba1-positive cells within the whole retina (Fig. 4E) showed a significant increase in *Glb1*^−/−^ mice compared to WT mice. Interestingly, no significant difference was observed between 4- and 7-month-old *Glb1*^−/−^ mice, indicating an early and consistent microglial response. Moreover, no significant difference was detected between young and old WT mice. Analysis of the distribution of Iba1-positive cells across the different retinal layers (Fig. 4F) revealed an increase of microglia/macrophages especially affecting the inner retinal layers (ganglion cell layer: GCL, inner plexiform layer: IPL) of *Glb1*^−/−^ mice with 7-month-old animals showing highest cell numbers, especially within the GCL. Only few Iba1-positive cells were detected in the outer retinal layers in all groups. In addition, the morphological phenotype of Iba1-positive cells was investigated (Fig. 5A–G). In *Glb1*^−/−^ mice, a significant increase in spiky and amoeboid Iba1-positive cells was observed (Fig. 5I,J). Interestingly, in contrast to the amoeboid morphology predominantly seen in the IPL adjacent to the GCL in 7-month-old animals, the OPL exhibited a significant increase in spiky Iba1-positive cells in 4 and 7-month-old *Glb1*^−/−^ mice (Fig. 5I,J), which suggests a gradual activation and migration of Iba1-positive cells towards the GCL. In contrast, ramified Iba1-positive cells were more abundant in the IPL and OPL of WT mice (Fig. 5H).

### Müller glial cells are not affected by intracytoplasmic storage of G_M1_-ganglioside

**Fig. 6:**
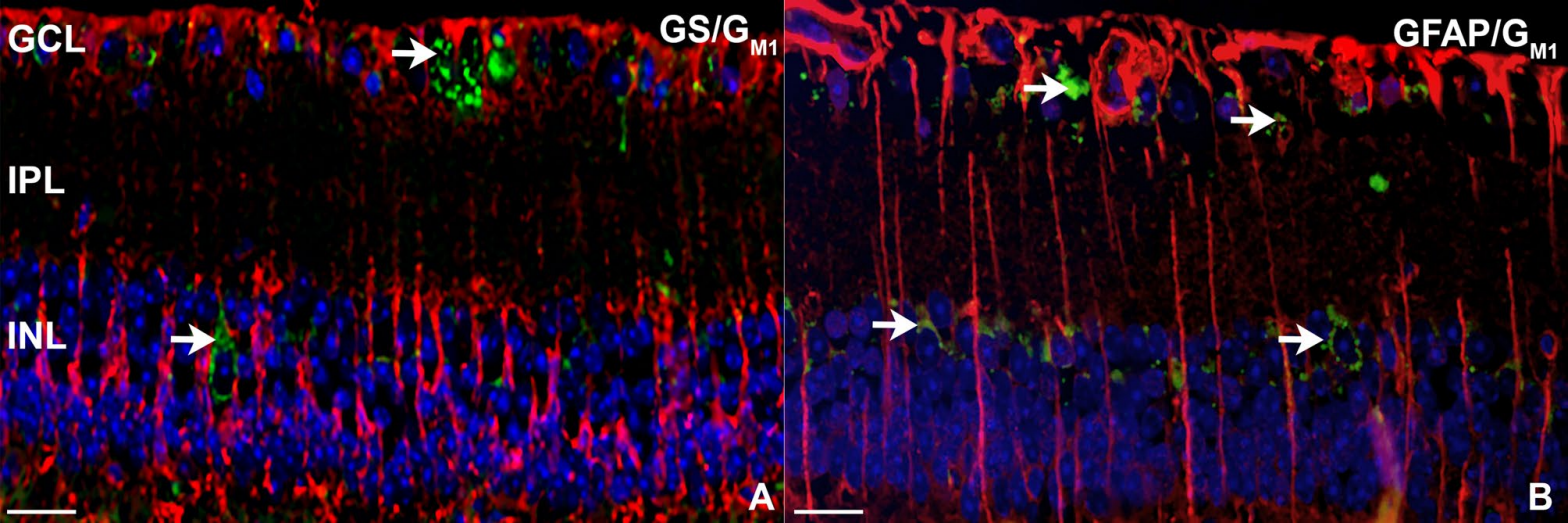
Immunofluorescence (IF) double staining of murine retinal tissue from a 7-month-old Glb1 knockout mouse. IF targeting G_M1_-1 shows an accumulation of GM1-positive lysosomal storage material (green; arrows) in ganglion cells (**A**,**B**). IF targeting glutamine synthetase (red, **A**) and GM1 (green) as well as glial fibrillary acidic protein (red, **B**) and GM1 (green) does not show colocalisation. This suggests that, in contrast to neurons of the RGC and INL (arrows), Müller cells are not affected by an excessive accumulation of storage material. Nuclei are counterstained with bisbenzimide (blue). Scale bars: 20 μm. GCL, ganglion cell layer; IPL, inner plexiform layer; INL, inner nuclear layer; OPL, outer plexiform layer.

**Fig. 7:**
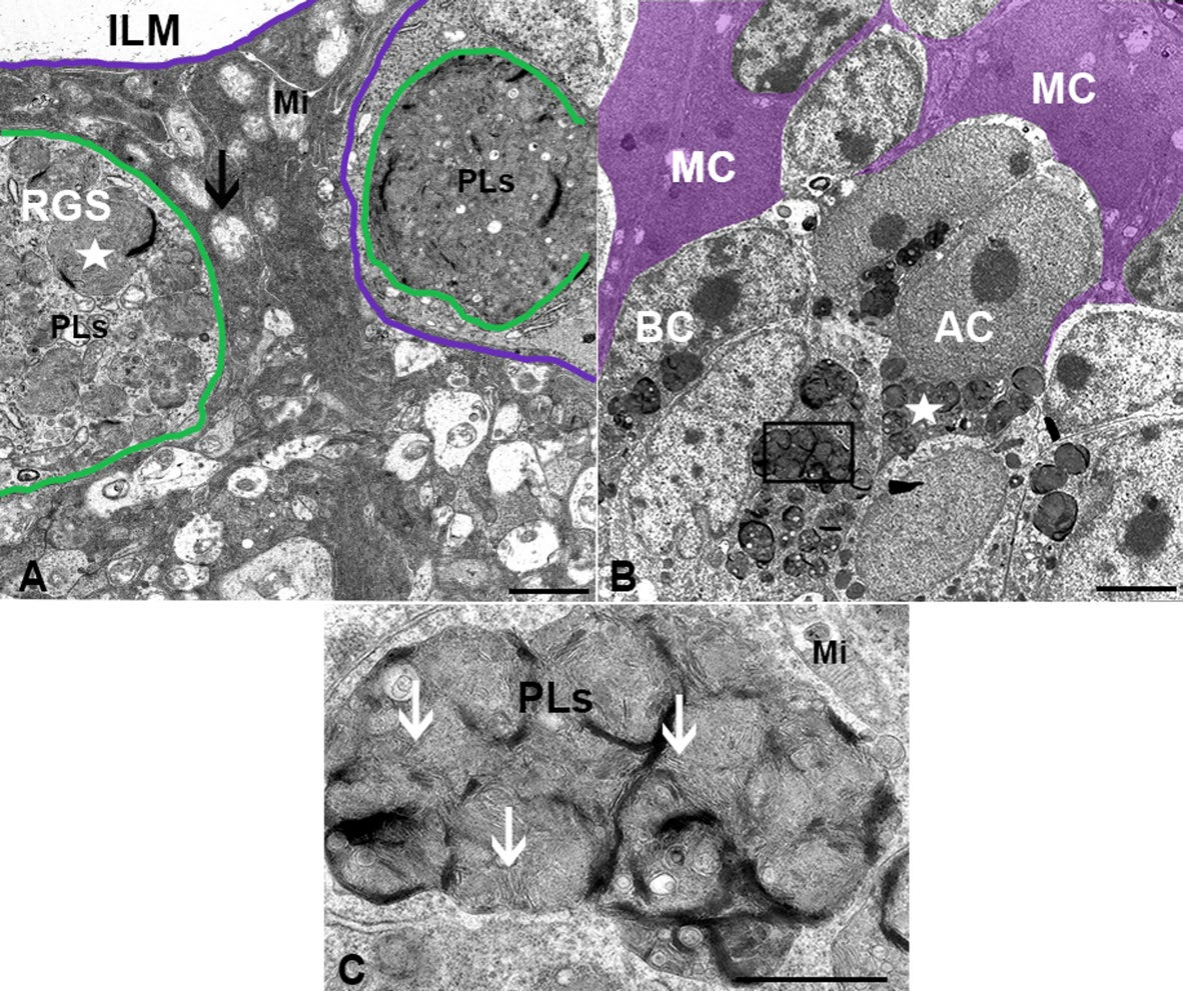
Transmission electron microscopic images illustrating the ultrastructural anatomy of the inner retinal tissue of a 7-month-old *Glb1* knockout (*Glb1*^−/−^) mouse. Müller cells (MCs) appear morphologically intact, with no evidence of storage material accumulation, while neurons show characteristic membranous inclusions of G_M1_ ganglioside. (**A**) The inner limiting membrane (ILM; outlined in purple) is formed by the “endfeet” of MCs. Müller cell endfeet (MCef) show mitochondrial (Mi) swelling (arrow). Retinal ganglion cells (RGC) and macrophages/microglia (M) show large phagolysosomes (PLs; outlined in green) with membranous inclusions (asterisk); bar: 1000 nm. (**B**) Amacrine cells (ACs) and bipolar cells (BCs) in the inner nuclear layer are tightly enveloped by the cell body of MCs (purple overlay ) and exhibit cytoplasmic membranous inclusions of G_M1_ gangliosides; bar: 2500 nm; insert: higher magnification in (**C**). (**C**) Higher magnification of a large phagolysosome with accumulation of electron dense, laminar, membranous inclusions of storage material resembling myelin figures or “zebra bodies” (white arrows); bar: 500 nm.

To further analyze the impact of G_M1_ ganglioside storage material accumulation on MCs reactivity, immunofluorescence double stainings combining anti-G_M1_ with GFAP or GS-staining were performed. The analysis confirmed the absence of detectable G_M1_ immunoreactivity in MCs, as no colocalisation of G_M1_ and either GFAP or GS was observed (Fig. 6A,B). In contrast, neurons in the GCL and INL displayed immunopositivity for G_M1_ (Fig. 6, arrows), indicating that storage material is primarily localized within neurons. In addition, transmission electron microscopic investigation of the retina of 7-month-old *Glb1*^−/−^ mice also revealed no indications for intracytoplasmic accumulation of G_M1_ storage material in MCs (Fig. 7). However, typical electron-dense, lamellated, concentric or whorled membranous inclusions of storage material, resembling myelin figures or ”zebra bodies” were clearly observed in neurons of RGC (Fig. 7A) and INL, as well as in both bipolar and amacrine cells in the INL (Fig. 7B,C). These findings emphasise the dominant susceptibility of neurons in the retina to G_M1_ accumulation.

## Discussion

The present study is the first to describe retinal changes in a late-onset murine model mimicking the adult form of G_M1_-gangliosidosis. Already at the age of 4 months, significant changes were found in the retina of knockout mice, especially with regard to the glial cell populations examined. Reactive glial changes within the retina can be categorized into “stereotypic” non-specific and specific responses^28,45^. The non-specific response is characterized by MC hypertrophy, proliferation, and upregulation of the intermediate filaments vimentin and GFAP^32,46,47^. In the present study, a significant and continuous increase of GFAP-positive MCs in *Glb1*^−/−^ mice was noted. This finding suggests activation in response to neuronal damage, especially since damage to the MCs themselves by intracytoplasmic accumulation of storage material could be excluded by immunofluorescence double staining and electron microscopy. Furthermore, an increase in GFAP-staining due to astrogliosis was excluded based on the cellular morphology of the counted cells and the localization of their nuclei within the INL. Interestingly, the upregulation of GFAP in MCs is consistent with previous observations in astrocytes and SGCs in animals of the same age in the same mouse model^17,34^. Similar to astrocytes in the brain and SGCs within DRG, MCs in the retina are able to upregulate GFAP expression in response to neuronal injury and dysfunction^26,48,49^. This indicates a universal mechanism of reactive gliosis, emphasizing a conserved glial response to neuronal ganglioside accumulation across diverse nervous compartments^49–51^. MC gliosis, as a common phenomenon in the pathogenesis of non-degenerative and degenerative retinopathies, is also considered a double-edged sword^28,47^. Initially, it supports neuroprotection and the release of neurotrophic factors and antioxidants, which favour neuronal survival and limit the extent of tissue damage. However, excessive gliosis can proceed into exuberant, long-lasting gliosis, which results in retinal scarring as well as increased neurotoxicity and neurodegeneration^27,52^. Few GFAP-positive MCs were also detected in 7-month-old WT mice, most likely indicating a certain degree of reactivity connected to age-related changes.

The specific response of MC activation is more targeted and can involve changes in GS expression, as demonstrated in the present study. Among retinal macroglia, MCs exhibit the highest levels of GS expression^36,53^. GS has a crucial function in the glutamate-glutamine cycle. GS regulates neurotransmitter metabolism by converting glutamate to glutamine, thereby maintaining a stable microenvironment for RGCs and preventing neuronal excitotoxicity. It is therefore continuously expressed in healthy retinal tissue^28,54–56^. In *Glb1*^−/−^ mice, the number of GS-positive MCs in 4-month-old *Glb1*^−/−^ mice was significantly increased compared to age-matched WT controls, most likely along with MC gliosis, as also indicated by GFAP-staining. However, in 7-month-old *Glb1*^−/−^ mice, the amount of GS-positive cells was comparable to WT counterparts. Notably, GS-positive MCs in 7-month-old *Glb1*^−/−^ mice were reduced compared to 4-month-old knockout mice. This result suggests a reduced expression of GS in MCs, which is most likely linked to the ongoing degeneration of RGC. Downregulation of GS expression by MCs has been previously reported after loss of major glutamate-releasing neurons, e.g., after inherited photoreceptor degeneration, retinal detachment, inflammatory and traumatic conditions, as well as in glaucoma in humans or other species^28,33,45^. The expression of GS in MCs is another common feature with astrocytes in the brain and SGC of the DRG, where GS also plays a key role in the removal of excess extracellular excitatory glutamate^57–59^. However, the exact pathogenic mechanism behind GS dysregulation in MCs in retinopathies and whether these changes contribute to visual impairment in the *Glb1*^−/−^ model require further investigation. The results obtained suggest that GFAP and GS represent suitable indicators for reactive, potentially noxious changes in MC activity and could therefore also be interesting pharmaceutical targets for intervention strategies.

Retinal changes in a transgenic mouse model of juvenile/early-onset G_M1_-gangliosidosis were characterized by the detection of storage material within ganglion cells^60^. Intracytoplasmic accumulation of lamellated storage material in swollen RGCs was also detected in affected calves and cats using electron microscopy, comparable to the results of the present study^11,61^. A significant reduction in RGCs was observed in 7-month-old *Glb1*^−/−^ mice, most likely as a result of excessive intracytoplasmic accumulation of G_M1_, as demonstrated using IHC. Loss of RGCs has also been observed in human patients with G_M1_-gangliosidosis as well as in various animal species, including cats, dogs and calves^19,22–25^. Within the context of G_M1_-gangliosidosis, ganglioside-mediated activation of the unfolded protein response could trigger RGC apoptosis^62^. Using IHC targeting cleaved caspase-3, a robust marker of apoptosis^63–65^, increasing numbers of cells with faint intranuclear labelling were observed. While procaspase-3 is located within the cytoplasm, active caspase-3 has been shown to be translocated into the nucleus^66^. A mildly increased number of cleaved caspase-3 positive cells was also detected in 7-month-old WT mice, suggesting an age-related phenomenon. Further insight into RGC integrity was gained through BRN3A immunostaining, a specific marker for RGCs^67^. Quantitative analysis revealed a significant reduction in BRN3A-positive cells in 7-month-old *Glb1*^−/−^ mice compared to all other groups. While BRN3A, a transcription factor, is not consistently and uniformly expressed in all RGC, its expression is known to decline in response to stress or early degeneration^67,68^. Studies have shown that it is downregulated in injured or degenerating neurons and that this precedes overt cell loss in some models. A decrease in BRN3A is also connected to sensory neuronal apoptosis [66, 68]. In the context of lysosomal storage disorders, transcriptional dysregulation due to substrate accumulation and cellular stress may contribute to neuronal vulnerability and apoptosis [62, 68]. Therefore, the observed reduction in BRN3A positive RGCs in *Glb1*^−/−^ mice likely reflects a combination of actual cell loss and downregulation of survival-promoting transcriptional programs. The nuclear caspase-3 staining, together with the decrease in BRN3A positive RGCs and the increase in amoeboid Iba-1 positive cells in vicinity of the RGCs, suggests apoptosis of retinal neurons in *Glb1*^−/−^ mice^66^. These findings further underscore the degenerative nature of retinal involvement in G_M1_-gangliosidosis.

Resident microglia in the retina also play a pivotal role in maintaining neuronal homeostasis. They are involved in immune surveillance and react quickly to retinal damage. However, a comprehensive characterization of the functions and tasks of retinal microglia is still incomplete in many areas [69]. In the present study, a significant increase in the total number of Iba1-positive cells was detected in *Glb1*^−/−^ mice at 4 and 7 months of age. This finding suggests an early and consistent activation of Iba1-positive cells during G_M1_-gangliosidosis. Under physiological conditions, Iba1-positive cells are predominantly located in the synaptic inner and outer plexiform layers (IPL and OPL) and are less frequently found within nuclear layers and around RGC and their axons^38^. Interestingly, in *Glb1*^−/−^ mice, not only an increase but also a spatial redistribution of Iba1-positive cells was detected. Previous studies in mouse and rat models for retinal degeneration reported migration of activated microglia throughout the retinal layers after photoreceptor loss^69,70^. In the present study, targeted migration towards the inner retinal layers was demonstrated, indicating a specific immune response towards neuronal damage and loss. In addition, morphological changes were observed, with microglia shifting from a ramified morphology to spiky and amoeboid phenotypes. A ramified to hyper-ramified morphology is thought to correlate with an inactivated functional state of microglia. Thin ramified processes allow continuous monitoring of the neuronal environment. Upon activation, the cells quickly adapt their morphology by shortening their processes and adopting a spiky or amoeboid phenotype with small pseudopodia, which enables rapid and dynamic migration to injury sites^26,39,40,73^. Compatible with this, higher numbers of spiky and amoeboid Iba1-positive cells were detected in the IPL and OPL of *Glb1*^−/−^ mice, again indicating a specific response towards neuronal damage and loss. Iba1-positive cells in retinal tissue of WT mice were primarily detected in IPL and OPL and predominantly exhibited a ramified morphology at both time points investigated.

The present study demonstrates that the *Glb1*^−/−^ mouse strain used in this investigation is suitable for characterizing the pathologic changes within the retina in a model of the adult form of G_M1_-gangliosidosis. The obtained results are consistent with changes described in bovine, feline, canine, and also human retinas^22–25^. Rodent models are highly valuable for research of retinal changes in G_M1_-gangliosidosis, especially due to wide range of possibilities of genetic manipulation^74,75^. However, species-specific differences and limitations, such as the sheer size of the eyeball in mice, must be considered when selecting suitable models. The cherry-red macula in G_M1_-gangliosidosis patients cannot be depicted in the listed animal models, as no comparable structure exists in animals other than primates.

Further studies including an assessment of visual capacity and a correlation with pathohistological and functional changes in retinal cells will be highly valuable. Furthermore, aditional research is needed to investigate the interactions between neurons and glial cells in order to explore the key pathophysiological mechanisms and develop ways to effectively alleviate the effects of the disease.

## Materials and methods

### Animals and tissue processing

WT and *Glb1*^−/−^ mice were sacrificed as part of an unrelated study. Briefly, *Glb1*^−/−^ mice were generated via insertion of a 636 bp fragment of the lacZ gene into the *Glb1* exon 15^17^. Mice were kept in ventilated cages on a 12 h light/dark cycle with ad libitum access to food and water. Breeding and genotyping was performed as described previously^17^. Ocular tissue samples were harvested from 5 male and female, 4- and 7-month-old WT and *Glb1*^−/−^ mice, respectively. During previous own studies using this mouse line, no sex-specific differences were obtained^17,76^. Mice were initially anaesthetized via intraperitoneal injection of medetomidine (Domitor^®^, Pfizer 1.0 mg/ml) and ketamine (Ketamin 10%, WDT, 100 mg/ml) diluted in NaCl at a dosage of 0.5 mg/kg medetomidine and 100 mg/kg ketamine, and subsequently euthanized by an intraperitoneal injection of an overdose of undiluted medetomidine and ketamine. Afterwards animals were transcardially perfused with phosphate-buffered saline (PBS), and the eye globes were carefully removed and fixed in 10% neutrally-buffered formalin for a maximum of 24 h. To provide comparable section levels for the subsequent investigations, fixed globes were sectioned vertically, with one half containing the optic nerve. Subsequently, tissue was embedded in paraffin wax (formalin-fixed and paraffin-embedded material; FFPE) with the cut surface facing downwards. The tissue was cut into approximately 3 μm thick serial sections using a microtome and subsequently mounted on SuperFrost-Plus^®^ slides (Thermo Fisher Scientific Inc., Fisher Scientific GmbH, Schwerte, Germany). For electron microscopy, eyes were fixed in 2.5% glutaraldehyde for at least 24 h.

### Ethics declaration

All animal experiments were performed in accordance with the German Animal Welfare Law and were approved by the local authorities (Niedersächsisches Landesamt für Verbraucherschutz und Lebensmittelsicherheit (LAVES), Oldenburg, Germany, permission number 33.8-42502-21/3632). This study is reported in accordance with the ARRIVE guidelines and conforms to its principles.

### Light and electron microscopy

Ocular sections were stained with hematoxylin and eosin (H&E) and examined using routine light microscopy. For transmission electron microscopy, samples were post-fixed with 1% osmium tetroxide and embedded in EPON 812 (Serva, Heidelberg, Germany). Semi-thin sections (ca. 0.5–1 μm-thick) were stained with toluidine blue and again investigated with a routine light microscope. 70 nm ultra-thin sections were cut with diamond knife (Diatome, Hatfield, USA) from selected regions of interest (ROI; from ILM till the end of the INL) and contrasted with uranyl acetate followed by lead citrate. Ultrastructural analyses were performed using a transmission electron microscope (Zeiss EM 906, Oberkochen, Germany) as previously described^77^.

### Immunohistochemistry and immunofluorescence

IHC on retinal sections was performed using primary antibodies targeting GS, GFAP, Iba1, βIII-tubulin (pan-neuronal marker), BRN3A (POU4F1; specific for GCL-neurons), cleaved caspase-3 and G_M1_-1. Briefly, IHC on FFPE tissue sections was performed using 0.5% H_2_O_2_ for 30 min to block endogenous peroxidase. For all antibodies applied, antigen retrieval was achieved by boiling for 20 min in citrate buffer (pH 6) or Tris-EDTA (BRN3A). To block non-specific binding sites, slides were incubated with 20% goat or horse (Iba1) serum diluted in PBS for 30 min. Subsequently, sections were incubated with the respective primary antibody (Table 1) overnight at 4 °C. The negative controls were incubated with inactivated serum of the host species of the primary antibody. Biotinylated goat-anti-rabbit IgG (BA-1000) or goat-anti-mouse IgG (BA-9200) diluted 1:200 (Vector Laboratories, Burlingame, CA, USA) were used as secondary antibodies. After visualization of the antigen-antibody reaction using the avidin-biotin-peroxidase complex (ABC) method (Vector Laboratories) and the chromogen 3,3′-diamino-benzidine (DAB), sections were counterstained with Mayer’s hematoxylin. For immunofluorescence (IF) staining, sections were also treated with H_2_O_2_ and incubated in citrate buffer (pH 6) for 20 min in a microwave. Afterwards, they were blocked with 20% goat serum in PBS containing 1% bovine serum albumin (BSA) and 0.1% Triton-X (Triton^®^ X-100, Merck Millipore, Merck KGaA). For single staining procedure, primary antibody targeting GFAP was incubated overnight at 4 °C. Negative controls were incubated with inactivated serum as described above. Secondary antibody (goat anti-rabbit Alexa Fluor 488; 1:200; 111-545-003; Jackson ImmunoResearch Europe Ltd) was applied at room temperature for 1 h. Specific markers were selected for double staining procedures (GFAP/G_M1_; GS/G_M1_). The first primary antibodies (GS; GFAP) followed by incubation of the second first antibody (G_M1_-1) were incubated overnight at 4 °C. Afterwards, an appropriate secondary antibody (goat anti-rabbit Alexa Fluor 488; 1:200; 111-545-003 and goat anti-mouse Alexa Fluor 488; 1:200; 115-545-003; Jackson ImmunoResearch Europe Ltd) was applied at room temperature for 1 h. To visualize the nuclei, bisbenzimide (diluted in double distilled water; bisbenzimide H 33258, Merck KGaA) was used, followed by mounting sections with a fluorescence mounting medium (Dako North America Inc.). Immunofluorescence images were taken with a Keyence BZ9000 fluorescent microscope (Keyence) with Nikon Plan Apo λ objectives (Nikon Europe BV)^77^.

**Table 1:** Primary antibodies used for immunohistochemistry (IHC) and Immunofluorescence (IF). FFPE, formalin-fixed, paraffin-embedded; GFAP, glial fibrillary acidic protein; GS, glutamine synthetase; Iba1, ionized calcium-binding adapter molecule 1; β-tubulin, βIII-tubulin; pc, polyclonal; mc, monoclonal.

Primary antibody	Clonality	Source	Pretreatment	Dilution	
				IHC-FFPE	IF-FFPE
Cleaved Caspase-3	mc rabbit	9661s, Cell Signalling Technology	Citrate buffer/800-Watt micowavve	1:200	–
GFAP	pc rabbit	Z0334, Dako North America Inc., Carpinteria, CA, USA		1:800	1:800
GS	pc rabbit	PA5-28940, Invitrogen, Thermo Fisher Scientific, Waltham, MA, USA		1:8000	1:8000
G_M1_-1	mouse hybridoma supernatant	SH30349, Developmental Studies Hybridoma Bank (DSHB); GM1-1 was deposited to the DSHB by Schnaar, R.L., University of Iowa, Iowa City, IA, USA		1:26	1:26
Iba1	pc goat	011-27991, FUJIFILM Wako Pure Chemical Corporation, Osaka, Japan		1:400	–
βIII-tubulin	pc rabbit	PRB-435P, E10344JE, Covance, USA		1:16,000	1:8000
BRN3A (POU4F1)	mc rabbit	EPR23257-285, ab245230, abcam, Amesterdam, Netherlands	Tris-EDTA/800-Watt micowavve	1:1000	–

### Tissue analysis, evaluation of cell density, and statistical analysis

Slides were digitalized using the Olympus VS200 slidescanner (Olympus Deutschland GmbH, Hamburg, Germany) to quantify immunopositive cells within different retinal tissue layers. The scanned slides were analyzed using QuPath for digital pathology image analysis^77^. Only intact, full-thickness retinal sections of good quality were chosen. For quantification of neurons with positive nuclear/cytoplasmic reaction for BRN3A or G_M1_-1, respectively, neurons of the GCL were counted manually on digitalized images. For evaluation of overall ganglion cell density, as well as quantification of GS and GFAP positive MCs, regions of interest (ROI; retinal tissue from ILM to the end of photoreceptor outer segment) were outlined manually for both immunostaining methods. Specifically, MCs immunopositively labelled for GFAP and GS were identified by their cell body in the INL and their radial processes extending the entire retinal thickness. Cell counts were determined manually in all ROIs, and only immunopositive cells with identifiable nuclei in the INL were included.

### Analysis of Iba1 cell distribution and morphological characteristics

For further quantification and examination of the distribution pattern of microglia/macrophages, Iba1 immunopositive cells were evaluated in all retinal layers. Only cells with clearly identifiable nuclei/soma were included. Additionally, the morphology of positive cells in each retinal layer was determined according to a previously published evaluation scheme^39,40^. Cells were categorized as ramified (”resting”), spiky (“activated”) or amoeboid (“activated”) according to their shape^39,40^. Cells categorized as ramified Iba1 immunolabelled cells were characterized by spider-like morphology with numerous linear processes and thin ramifications as well as clear borders of the soma. In contrast, Iba1-positive cells displaying shortened processes (larger near the soma and smaller in the periphery) and different diameters were interpreted as spiky cells. In addition, cells with round morphology and very short processes or even without processes, displaying an increased diameter of the soma larger than that of the spiky cells, were categorized as amoeboid cells.

### Statistical analysis

Statistical data analysis was performed using SPSS for Windows (version 29.0.1.0; IBM^®^ SPSS^®^ Statistics, SPSS Inc., Chicago, IL, USA). Normal distribution was tested via Kolmogorov–Smirnov and Shapiro–Wilk tests. Differences between individual groups were analyzed via the Kruskal–Wallis test followed by Dunn’s post-hoc tests and adjustment for multiple testing using the Bonferroni procedure. Statistical significance was accepted at a p-value of < 0.05.

## Data Availability

The data are available from the corresponding author upon request.
